# Special Issue “Molecular Immunology of Solid Tumors, 2nd Edition”

**DOI:** 10.3390/ijms26157329

**Published:** 2025-07-29

**Authors:** Steven Fiering

**Affiliations:** Dartmouth Geisel School of Medicine, Dartmouth Cancer Center, Hanover, NH 03756, USA; fiering@dartmouth.edu

There has been an intellectual, technical, and clinical revolution in understanding and utilizing the interaction of tumors and immune cells. Prior to the turn of this century, tumor immunology was a niche field that was populated by a small number of passionate scientists, while oncologists and cancer biologists generally ignored what were very compelling mouse studies. We now understand that immune cells are an integral part of solid tumors, but their influence on tumors is complex and variable. The pro- and anti-tumor importance of immune cells of many types is now well established in solid tumor biology, as is the potential to treat cancer using immune-based therapies. The intellectual aspect of this revolution is documented in Figure 1, which presents annual PubMed-indexed publications that include “tumor” and “immune” in the abstract. As is obvious, tumor immunology has gone from a very quiet specialty field to a huge aspect of cancer research. Tumor immunology is a major new area of intellectual and clinical research in cancer, has improved outcomes for many, and will lead to much more dramatic and broadly improved patient outcomes. This, not surprisingly, has been paralleled by an explosion of interest in the tumor microenvironment, illustrated in Figure 2. We have gone from a focus on the tumor cell exclusively to a major focus on the interaction of the many normal and tumor cells that make up the tumor. The realization is established that tumors are not single rogue cell types, but rather aberrant cells directing the complex interplay of other normal cells, all of which occurs in response to what is essentially natural selection. 

At this point, it is clear that cancer cannot be understood without accounting for immune cells and how they interact with tumors and tumor-influenced stromal cells. Despite clear progress in cancer immunotherapy, which has benefited many patients, most patients do not respond to current immunotherapies, primarily a limited number of checkpoint-blocking antibodies that stimulate T cells. The field requires a deeper and broader understanding of how immune cells support or oppose cancer development and the molecular pathways involved in order to create the intellectual foundation for the next generation of cancer immune therapies. This understanding is being generated and disseminated rapidly by academic and commercial researchers. The quantity of publications, one measure of intellectual investment, reflects that investment, as shown by the tables. 

This Special Issue on the molecular immunology of solid tumors will publish research to increase our understanding of immune cell interactions with tumors and the molecular pathways that mediate those interactions. Cancer is a highly complex disease and the huge variety of cancers, many with myriad subtypes, compounds that complexity. This Special Issue on the molecular immunology of solid tumors reflects that complexity. As with the story of the blind men touching and describing different parts of an elephant, the many relevant publications and the knowledge contributed here are slowly generating an accurate, and most importantly, actionable understanding of how immune cells both support and oppose the tumors.

The six papers in the Special Issue reflect the breadth and complexity of topics relevant to the immunology of solid tumors. For Contribution 1, Hargrove-Wiley et al. explore immune biology associated with the poorer outcomes in men with breast cancer as compared to women with the disease. This study shows that a sex-dependent immune response is a contributor to poorer outcomes. For Contribution 2, Barrano et al. report on studies of people’s pets with spontaneous canine inflammatory mammary cancer, treated with an immunostimulatory plant virus as intratumoral immunotherapy. The notable efficacy of the treatment was previously reported. This manuscript reports on transcriptomic changes and focuses on increased neutrophil-recruiting cytokines and increased neutrophil recruitment after treatment, an interesting result since increasingly neutrophils are recognized as having both suppressive and stimulatory immune influence in solid tumors. For Contribution 3, Pizarro et al. present a retrospective study investigating immune characteristics in 124 patients with early-stage high-grade serous ovarian cancer (HGSOC). It is concluded that increased intraepithelial CD8+ T cells as well as higher CD8/CD4 ratios in the tumors are associated with better outcomes. For Contribution 4, Milchram et al. studied serum immunoglobulin in pre- and post-surgery patients with either glioblastoma or meningioma to catalog tumor-associated antigen recognition. The report notes that many different proteins, often associated with signaling pathways, were recognized. There were 10–25% similar antigens in pre- vs post-operative situations and in glioblastoma vs meningioma comparisons. For Contribution 5, Mielcarska et al. performed a meta-analysis review of how B7-H3 expression in solid tumors correlates with patient outcomes. Overall, 51 studies and 11,135 patients were included in the meta-analysis which concluded that high B7H3 expression is a negative prognostic indicator for patient survival. For Contribution 6, Mandal et al. reviewed the rapidly growing literature on the sympathetic nervous system influences on cancer, with a focus on hepatocellular carcinoma. They concluded with a discussion of potential nervous system-focused interventions to treat liver cancer. 

The goal of the work is understanding in detail how the immune system interacts with cancer and developing new immunotherapies that will further extend survival of cancer patients. This work is proceeding, and the pace of discovery is increasing, with the result that the ancient scourge of cancer is now being understood on the molecular level. Improved understanding of immune characteristics of solid tumors will generate novel immune-based treatments, and the length and quality of life of cancer patients will undoubtedly improve as a consequence.

## Figures and Tables

**Figure 1:**
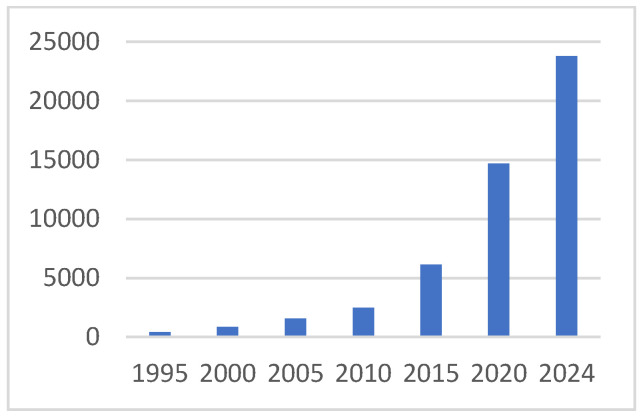
Annual publications of papers with “tumor” and “immune” in abstract.

**Figure 2:**
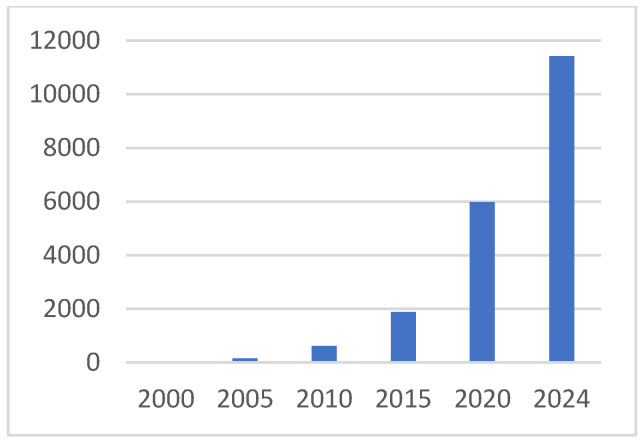
Annual publications of papers with “tumor microenvironment” in abstract.

